# Supplementary material for: Transcriptional Profiling of Non-injured Nociceptors After Spinal Cord Injury Reveals Diverse Molecular Changes
Source: Front Mol Neurosci. 2019 Nov 26;12:284. doi: 10.3389/fnmol.2019.00284 (PMC6988781; doi:10.3389/fnmol.2019.00284)
Supplement: Supplementary file 1 [file Data_Sheet_1.docx]

**Transcriptional profiling of non-injured nociceptors after spinal cord injury reveals diverse molecular changes**

Jessica R. Yasko^1^, Isaac L. Moss^2^, Richard E. Mains^1,*^

^1^ University of Connecticut Health Center, Department of Neuroscience, Farmington CT 06030

^2^ University of Connecticut Health Center, Department of Orthopedic Surgery and the Comprehensive Spine Center, Farmington CT 06030

^*^  mains@uchc.edu 860-679-8894

**Supplementary Figure S1**

**Supplementary Figure 1. Mobility, cytokine controls.** (A) Open field behavior (10-minute trials) conducted on naïve and sham mice 0,1,3,5 and 7 days post-surgery does not differ significantly at any time point in time spent in periphery, N=6 each. Testing done 1 day post-SCI also does not differ significantly in time spent in periphery, N=6 naïve, N=6 sham, N=4 SCI. (B) Cytokine ELISAs on spinal cord segments at the level of laminectomy (T8-T11) show no significant differences between naïve and sham mice 5 or 7 days post-surgery.

**Supplementary Figure S2**

**Supplementary Figure 2. Volcano plots, Venn diagram.** (A) Volcano plot of RNAseq transcript p-Values calculated by DESeq2 comparing SCI vs. naïve, SCI vs. sham, or (B) sham vs. naïve conditions, RPKM >10. (C) Venn diagram of statistically significant genes from the RNAseq data set determined by an overlap of DESeq2 significant genes (p<0.05) and outlier removal, with a cutoff excluding RPKM values <10.

**Supplementary Figure S3**

**Supplementary Figure 3. Ion channel heatmaps.** (A) Acid sensing ion channels (Asics), (B) calcium channels, (C) glycine receptors, and (D, E) purinergic receptors (P2Y, P2X). Expression patterns are similar across all three conditions. Despite their known relevance in pain transduction, no significant changes were observed at the 4 day time point tested. RPKM <1 were not included.

**Supplementary Figure S4**

**Supplementary Figure 4. Transcription factor heatmap.** Significant changes between SCI vs. naïve or SCI vs. sham conditions by DESeq2: Ahr, Atf4, Cpeb1, Creb3l1, Csrnp3, Drap1, Egr1, Erf, Foxn3, Irf5, Jun, Junb, Mafg, Mef2c, Meis3, Myc, Nr3c1, Nr4a1, Pbx1, Tbx3, Tet1, Zfhx3, Zfp28, Zfp30, Zfp41, Zkscan8, Zscan22. RPKM <1 were not included.

**Supplementary Figure S5**

**Supplementary Figure 5. Transcription factors.** Transcript levels that significantly differ 4 days post-SCI. DESeq2 p-Value based on SCI vs Naïve or SCI vs Sham comparisons. P-Values that are not listed were >0.05.

**Supplementary Figure S6**

**Supplementary Figure 6. Primer list.** Primers for voltage-gated channels, receptors, Trp channels, or involvement in the synaptogenesis pathway were designed for PCR products 111-143 bp in size, Tm=59.5-63.5C, validated on whole DRG tissue before preamplification. Hprt was the least variable gene based on RNAseq results, * indicates housekeeping gene.

**Supplementary Figure S7**

**Supplementary Figure 7.** Proposed model of how this signaling pathway may be contributing to the onset of chronic pain 4 days post-SCI in DRG distal to the site of injury. RNAseq data, IPA analysis, and qPCR validation suggest Ntrk2 (TrkB) signaling may play a role during the transition from acute to chronic pain at 4 days post-SCI.
